# Involvement of Mitochondrial Dysfunction in the Inflammatory Response in Human Mesothelial Cells from Peritoneal Dialysis Effluent

**DOI:** 10.3390/antiox11112184

**Published:** 2022-11-04

**Authors:** Olalla Ramil-Gómez, Mirian López-Pardo, Jennifer Adriana Fernández-Rodríguez, Ana Rodríguez-Carmona, Teresa Pérez-López, Carlos Vaamonde-García, Miguel Pérez-Fontán, María José López-Armada

**Affiliations:** 1 Aging and Inflammation Research Laboratory, Institute for Biomedical Research of A Coruña (INIBIC), 15006 A Coruña, Spain; 2 Endocrine, Nutritional and Metabolic Diseases Group, Faculty of Health Sciences, A Coruña, INIBIC, 15006 A Coruña, Spain; 3 Nephrology & Infectious Diseases Group, Institute of Research and Innovation in Health (i3s), 4200-135 Porto, Portugal; 4 Congenital and Structural Heart Disease, INIBIC, 15006 A Coruña, Spain; 5 Division of Nephrology, University Hospital A Coruña (CHUAC), 15006 A Coruña, Spain; 6 Rheumatology Research Group, INIBIC, CHUAC, 15006 A Coruña, Spain

**Keywords:** peritoneal dialysis, mitochondria, oxidative stress, inflammation, mesothelial cells, resveratrol

## Abstract

Recent studies have related mitochondrial impairment with peritoneal membrane damage during peritoneal dialysis (PD) therapy. Here, we assessed the involvement of mitochondrial dysfunction in the inflammatory response in human mesothelial cells, a hallmark in the pathogenesis of PD-related peritoneal membrane damage. Our ex vivo studies showed that IL-1β causes a drop in the mitochondrial membrane potential in cells from peritoneal effluent. Moreover, when mitochondrial damage was induced by inhibitors of mitochondrial function, a low-grade inflammatory response was generated. Interestingly, mitochondrial damage sensitized mesothelial cells, causing a significant increase in the inflammatory response induced by cytokines, in which ROS generation and NF-κB activation appear to be involved, since inflammation was counteracted by both mitoTEMPO (mitochondrial ROS scavenger) and BAY-117085 (NF-κB inhibitor). Furthermore, the natural anti-inflammatory antioxidant resveratrol significantly attenuated the inflammatory response, by reversing the decline in mitochondrial membrane potential and decreasing the expression of IL-8, COX-2 and PGE_2_ caused by IL-1β. These findings suggest that IL-1β regulates mitochondrial function in mesothelial cells and that mitochondrial dysfunction could induce an inflammatory scenario that sensitizes these cells, causing significant amplification of the inflammatory response induced by cytokines. Resveratrol may represent a promising strategy in controlling the mesothelial inflammatory response to PD.

## 1. Introduction

Peritoneal dialysis (PD) is a growing therapeutic alternative for end-stage kidney disease. In this procedure, the peritoneum of the patient is used as a semipermeable membrane, which allows selective transport of water and solutes to and from the organism, favored by natural or artificially generated concentration gradients between the blood and the dialysis solution introduced in the peritoneal cavity [1]. However, in long-term PD, dialysis fluids features (such as acidic pH, high glucose concentration, glucose degradation and advanced glycation end-products) along with the presence of a permanent intraperitoneal catheter, can induce progressive peritoneal injury and a consequent loss of the functional characteristics of the membrane [2]. The pathogenesis of this disorder is complex, but extensive evidence supports the role of mesothelial cells (MCs) in the structural and functional alterations of the peritoneum during PD. The monolayer of MCs that coat the peritoneum represents the first line of contact with PD solution [3,4].

Mitochondria are highly dynamic cellular organelles that harbor multiple functions [5]. One of their primary competencies is to produce cellular energy (adenosine triphosphate, ATP) by oxidative phosphorylation, generating a proton gradient across the inner membrane that defines the mitochondrial membrane potential (ΔΨm), a key indicator of mitochondrial activity. Because of their activity, mitochondria are the most important source of reactive oxygen species (ROS), and remarkably, they are also targets of these molecules. Under physiological conditions, ROS have many cellular functions [6]; by contrast, in a mitochondrial impairment scenario, there is a boosted production of ROS, which could induce an inflammatory response [7]. Another key event is the high mutation rate associated with mtDNA that is mainly due to the action of ROS production [8,9]. Furthermore, oxidative damage to mitochondria is often associated with damaged proteins, membrane lipids and mtDNA [10], which can also increase mitochondrial damage, leading to a vicious cycle [5]. This is the reason why mitochondrial impairment has been linked to several human disorders and pathological pathways [11,12,13,14,15].

Regarding the case of PD, several studies from our group and also from other authors have recently demonstrated that mitochondrial dysfunction plays a relevant role in the pathophysiology of the peritoneal membrane disorders induced by PD [13,16]. Our ex vivo results provide clear evidence that mitochondrial damage promotes the disruption of the MC monolayer affecting the structure of the peritoneal membrane during the course of PD and that mtDNA levels measured in the effluent dialysate of PD patients could perform as a biomarker of PD-induced damage to the peritoneal membrane [13]. In addition, mitochondrial impairment is one of the main promoters of glucose-rich PD fluid-induced mtROS, apoptosis, and mtDNA damage in human peritoneal mesothelial cells [17,18], as well as T-cell interleukin (IL)-17 differentiation that induces the inflammatory response in MCs [19]. All these processes contribute to the disorder of peritoneal membrane function and, as a consequence, to PD technique failure, as often found in long-term PD patients. However, our understanding of these phenomena is incomplete, and further studies are needed to understand how mitochondrial damage could be involved in the structural and functional alterations of the peritoneum during PD and how we could successfully prevent this progressive peritoneal damage that eventually causes loss of dialysis function.

The purpose of the present study was to determine the role of mitochondrial dysfunction in the development of inflammation and angiogenesis by human MCs and to evaluate whether mitochondrial dysfunction could intensify the cytokine-induced expression of inflammatory and angiogenic mediators in these cells. Due to their role in the pathophysiology of peritoneal membranes during PD, we decided to examine IL-8 and cyclooxygenase (COX)-2. Furthermore, we examined the effect of resveratrol, a natural polyphenol of plant origin, on the aforementioned processes.

## 2. Materials and Methods

### 2.1. Ethics Statement

The research was approved by the local Ethics Committee (Galicia, Spain) (code number 2014/454) and carried out according to the Spanish Law for Biomedical Research (Law 14/2007-3 of July) and the Helsinki Declaration (2008) of the World Medical Association. Written informed consent was obtained from all patients who participated in the study and the samples obtained were coded to maintain anonymity. 

### 2.2. Cell Culture and Stimulation

Primary mesothelial cells were isolated from the peritoneal effluent of dialysis patients by centrifugation at 1500 rpm for 10 min (min). The cells obtained were seeded in 48-well Costar plates (Corning, Kennebunk, ME, USA) and cultured in Roswell Park Memorial Institute (RPMI) medium (Lonza, Basel, BL, Switzerland) supplemented with 20% heat-inactivated fetal bovine serum (FBS) (Gibco, Paisley, INH, UK), 5000 U/mL penicillin (Gibco), 5000 µg/mL streptomycin (Gibco) and 0.12 U/mL human insulin (Novo Nordisk, Bagsvaerd, CPH, Denmark). After reaching 80% of confluence, cells with an epithelial phenotype were selected to perform the experiments. Cells were maintained in RPMI supplemented with 0.5% FBS 24 h (h) before performing the experiments and during cell stimulation. 

Mesothelial cell line Met5A (ATCC, reference CRL-9444) was cultured in Dulbecco’s Modified Eagle Medium (DMEM) (Lonza) supplemented with 10% heat-inactivated FBS, 5000 U/mL penicillin, 5000 µg/mL streptomycin and 0.12 U/mL human insulin. Cells were seeded in 12-well Costar plates (Corning) and after reaching 80% of confluence, complete medium was replaced by DMEM 0.5% FBS for 48 h. Thereafter, experiments were carried out in DMEM without FBS.

Cytokines IL-1β (Sigma-Aldrich, St. Louis, MO, USA) and TNFα (R&D systems, McKinley Place, MN, USA) were used as inductors of the inflammatory response. Mitochondrial dysfunction was induced using the ATP synthase inhibitor oligomycin (OLI, Sigma-Aldrich) and the pro-oxidant methyl viologen dichloride (paraquat-PQ, Sigma-Aldrich) [15]. Carbonyl cyanide 4-trifluoromethoxy-phenylhydrazone (FCCP, Sigma-Aldrich) was used as a mitochondrial oxidative phosphorylation uncoupler, and BAY-117085 (Sigma-Aldrich) as an inhibitor of the activation of nuclear factor-κB (NF-κB) pathway. The selective ROS scavenger mitoTEMPO (Santa Cruz Biotechnology; Santa Cruz, CA, USA) and resveratrol (Sigma-Aldrich) were also tested. After checking cell viability with different doses of resveratrol and verifying that it does not have cytotoxic effects (data not shown), a concentration of 5 µM was chosen, as previous studies showed evidence of its antioxidant and anti-fibrotic properties [20].

### 2.3. Mitochondrial Membrane Potential Assessment

Mitochondrial membrane potential was determined by flow cytometry using the fluorescent red-orange dye tetramethylrhodamine (TMRM; ThermoFisher scientific, Orlando, FL, USA), whose signal is proportional to the membrane potential. Mesothelial cells were incubated with TMRM (250 nM) in complete culture medium for 30 min and were trypsinized, washed with phosphate-buffered saline (PBS, Gibco), and centrifuged at 1500 rpm for 10 min. The pellet obtained was resuspended in 200 µL of PBS and fluorescence was measured using the FACScalibur cytometer (Becton Dickinson, Mountain View, CA, USA). The data obtained were analyzed with the software CellQuestPro 5.1 (BD). Mitochondrial depolarizer FCCP (5 µM, 30 min) was used as the technical positive control.

### 2.4. Determination of COX-2 and IL-8 mRNA Expression

To isolate mRNA, cell lysis was carried out using Trizol reagent (Invitrogen, Waltham, MA, USA). Briefly, cells were washed with PBS, incubated with 200 µL of Trizol for 5 min and transferred to an RNase-free Eppendorf tube. Then, the obtained lysate was mixed with 40 µL of chloroform (Honeywell, Frankfurter, HE, Germany) and incubated at room temperature for 3 min. After centrifugation at 12,000× *g* for 15 min at 4 °C, the aqueous phase was collected, mixed with 100 µL of isopropanol to precipitate the RNA, and centrifuged at 12,000× *g* for 10 min at 4 °C. The supernatant was discarded, and the pellet was washed twice in increasing concentrations of alcohol to purify the RNA. After centrifugation, the supernatant was removed, and the pellet was dried at room temperature and resuspended in warm RNase-free water. Afterwards, it was incubated at 55 °C for 10 min and RNA was quantified using a Nanodrop ND-1000 spectrophotometer (ThermoFisher scientific). Next, 1 µg of RNA was treated with Dnase I (Invitrogen), and retrotranscribed to cDNA with the SuperScript VILO Master Mix kit (ThermoFisher), according to the manufacturer’s protocol. Real-time reverse transcription polymerase chain reaction (realtime RT-PCR) was performed by mixing 4 µL of cDNA with 5 µL of Lightchycler 480 probes master (Roche, Indianapolis, IN, USA), 0.5 µL of forward and reverse primers (20 µM) (Roche), 0.1 µL of Taqman UPL probes (Roche) and 0.2 µL of Rnase-free water. Amplification and fluorescence detection were carried out using the LightCycler 480 II Instrument (Roche), where the samples were incubated as follows: 95 °C for 10 min, 45 cycles of 95 °C for 10 s (s), 60 °C for 30 s and 72 °C for 1 s; and 1 final cycle of 40 °C for 20 s. Real-time RT-PCR reactions were performed in duplicate, and cyclooxygenase 2 (COX-2) (oligonucleotides: forward 5′-cttcacgcatcagtttttcaag-3′, reverse 5′- tcaccgtaaatatgatttaagtccac-3′ and probe 23) and interleukin 8 (IL-8) (oligonucleotides: forward 5′-gagcactccataaggcacaaa-3′, reverse 5′-atggttccttccggtggt -3′ and probe 72) expression was normalized using TATA box-binding protein 1 (TBP1) (oligonucleotides: forward 5′-gcccatagtgatctttgcagt-3′, reverse 5′-cgctggaactcgtctcacta-3′ and probe 67) as a housekeeping gene. Data are expressed as fold changes, with respect to the basal expression.

### 2.5. PGE_2_ and IL-8 Assays

The levels of released prostaglandin E2 (PGE2) and IL-8 were measured using commercial ELISA kits (Cayman chemical, Ellsworth Rd, MI, USA and R&D systems, respectively), following the manufacturer’s instructions. Data are expressed as picograms/mL. 

### 2.6. Immunofluorescence Detection of p65

Cells were cultured in 8-well glass (Merk Millipore, Cork, COR, Ireland). After been stimulated, cells were fixed for 30 min with 4% paraformaldehyde at 4 °C, washed in PBS and permeabilized with a PBS solution containing 0.5% Triton X-100 for 10 min, followed by another incubation period of 30 min in PBS with 0.5% Triton and 0.2% BSA at room temperature. Then, cells were incubated with a rabbit polyclonal anti-p65 antibody (#F0514, Santa Cruz Biotechnology) for 2 h at room temperature, washed with PBS with 0.5% Triton and incubated with Alexa Fluor 568-conjugated donkey anti-rabbit IgG (#A10042, Invitrogen) for 1 h at room temperature. The cells were then washed and counterstained with DAPI. Images were taken using an Olympus BX61 microscope (Olympus, Tokyo, Japan) and further analyzed with ImageJ software version 1.50 e (National Institutes of Health, Bethesda, WA, USA). Fluorescence intensity of the red channels across individual cells (n = 30 per condition) was obtained using 200 × 50 px transects. To obtain plot profiles, a baseline correction was applied using GraphPad Prism 5.01 software, which defined the baseline as the average of the first 15 and last 15 values of the transect, and performed the calculation as the difference between the original value and baseline. 

### 2.7. Statistical Analysis

Statistical analysis was performed using GraphPad PRISM software version 9 (GraphPad Software, La Jolla, CA, USA). The data were presented as the mean ± SEM or as representative results, as indicated. The Kolmogorov–Smirnov test was used to analyze the data distribution. When the data showed a normal distribution, a paired *t*-test was performed, and non-parametric data were analyzed using the Mann–Whitney test. The differences were considered significant when the *p* value ≤ 0.05. 

## 3. Results

### 3.1. Involvement of Mitochondrial Dysfunction in the Inflammatory Response of Human Mesothelial Cells

**Figure 1:**
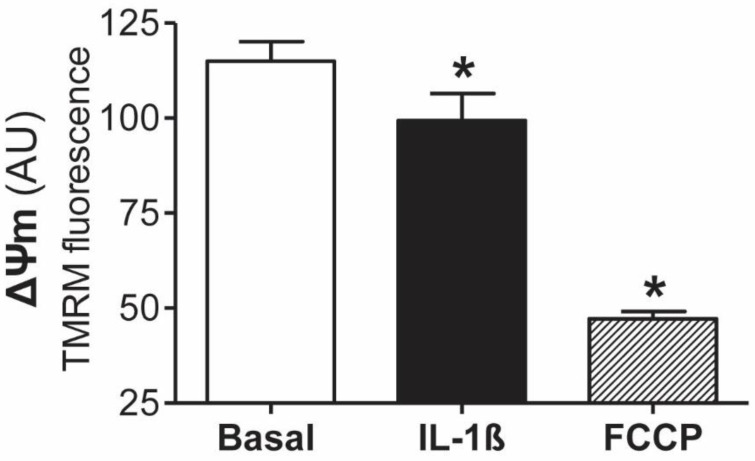
IL-1β induces mitochondrial depolarization in effluent-derived human mesothelial cells. Mitochondrial depolarization was assayed in mesothelial cells with epithelial phenotypes derived from peritoneal equilibration test (PET) samples stimulated for 12 h with IL-1β and loaded with tetramethylrhodamine methyl ester (TMRM) for 30 min before the end of incubation. Fluorescence quantification for TMRM (n = 7) is expressed as the mean ± SEM of fluorescence intensity in arbitrary units (AU). * *p* ≤ 0.05 vs. basal.

We tested the hypothesis that mitochondrial dysfunction is involved in the inflammatory response of human peritoneal mesothelial cells. The first set of experiments was focused on studying the effects of IL-1β on mitochondrial function by assessment of mitochondrial membrane potential (ΔΨm). The increase in IL-1β in peritoneal dialysis effluent (PDE) is an early event in short-term PD patients [21]. As shown in Figure 1, our ex vivo studies showed that IL-1β (1 ng/mL) causes a significant (*p* ≤ 0.05) drop in the ΔΨm in mesothelial cells from peritoneal effluent with epithelial phenotypes, as assessed by tetramethylrhodamine methyl ester staining. Mitochondrial uncoupler carbonyl cyanide 4-trifluoromethoxy-phenylhydrazone (FCCP) was used as a positive control of the technique, inducing a decrease of 58.9% in fluorescence compared to the basal condition. 

Then, to study the repercussion of this mitochondrial impairment in the inflammatory response, we evaluated the effect of mitochondrial dysfunction on the expression of common inflammatory mediators in mesothelial cells. To assess this response, cells were exposed to oligomycin or paraquat, two classical mitochondrial damage inducers. As a result, mitochondrial dysfunction induced by oligomycin provoked an increase in IL-8 and COX-2 mRNA expression at 6 and 18 h of stimulation that did not achieve statistical significance. On the other hand, in a time-dependent manner, paraquat induced a significant rise in IL-8 expression, which plays a crucial role in attracting immune cells during the inflammatory response (see Supplementary Figure S1). IL-1β (5 ng/mL) was used as a positive control, reaching values of 75.7 ± 20.6 and 65.2 ± 29.8 of IL-8 and COX-2 mRNA expression, respectively, compared to the basal condition at 6 h. These results show that mitochondrial dysfunction can trigger a low level of inflammatory response in mesothelial cells. 

### 3.2. Mitochondrial Dysfunction Synergizes with Inflammatory Cytokines to Aggravate the Inflammatory Response in Human Mesothelial Cells 

**Figure 2:**
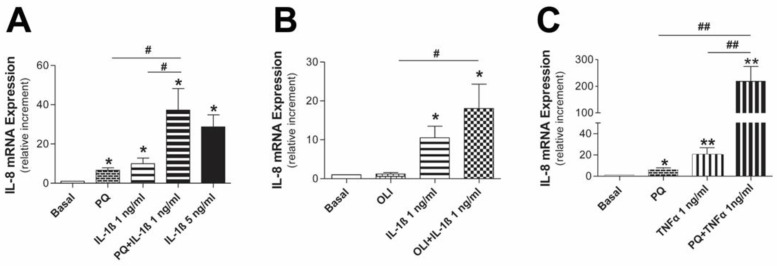
Mitochondrial dysfunction modulates IL-8 mRNA expression induced by cytokines in human mesothelial cells. Met5A cells were preincubated with the selective mitochondrial respiratory chain inhibitors paraquat (PQ; 1 mM) or oligomycin (OLI; 10 µg/mL) for 30 min before treatment with IL-1β (1 ng/mL, 6 h) or TNFα (1 ng/mL, 18 h). IL-8 mRNA expression for (**A**) PQ + IL-1β (n = 5), (**B**) OLI + IL-1β (n = 7) and (**C**) PQ + TNFα (n = 4) is expressed as the mean ± SEM fold change vs. basal condition. * *p* ≤ 0.05 and ** *p* ≤ 0.01 vs. basal, and ^#^ *p* ≤ 0.05 and ^##^ *p* ≤ 0.01.

Continuous exposure to PDF causes a low-grade inflammatory state in the peritoneum that can lead to the epithelial-to-mesenchymal transition (EMT) process during PD treatment. In this regard, recent studies by our group have shown that mesothelial cells suffer from mitochondrial dysfunction during EMT that occurs in the course of PD treatment [13]. Based on these premises, we evaluated if mitochondrial dysfunction synergizes with inflammatory cytokines to induce a higher inflammatory response in human mesothelial cells. Our results showed that in Met5A combined stimulation, mitochondrial inhibitors and inflammatory cytokines caused a significant rise in IL-8 mRNA expression compared to their separate stimulation in all cases (Figure 2). Thus, the highest expression of IL-8 was obtained with the treatment of paraquat and TNFα, achieving an effect ~10 times greater than that obtained with the inflammatory cytokine alone (Figure 2C). The doses and stimulation time were selected after evaluating concentration–time curves for every treatment (data not shown), taking our previous experience and literature information into account [22,23].

**Figure 3:**
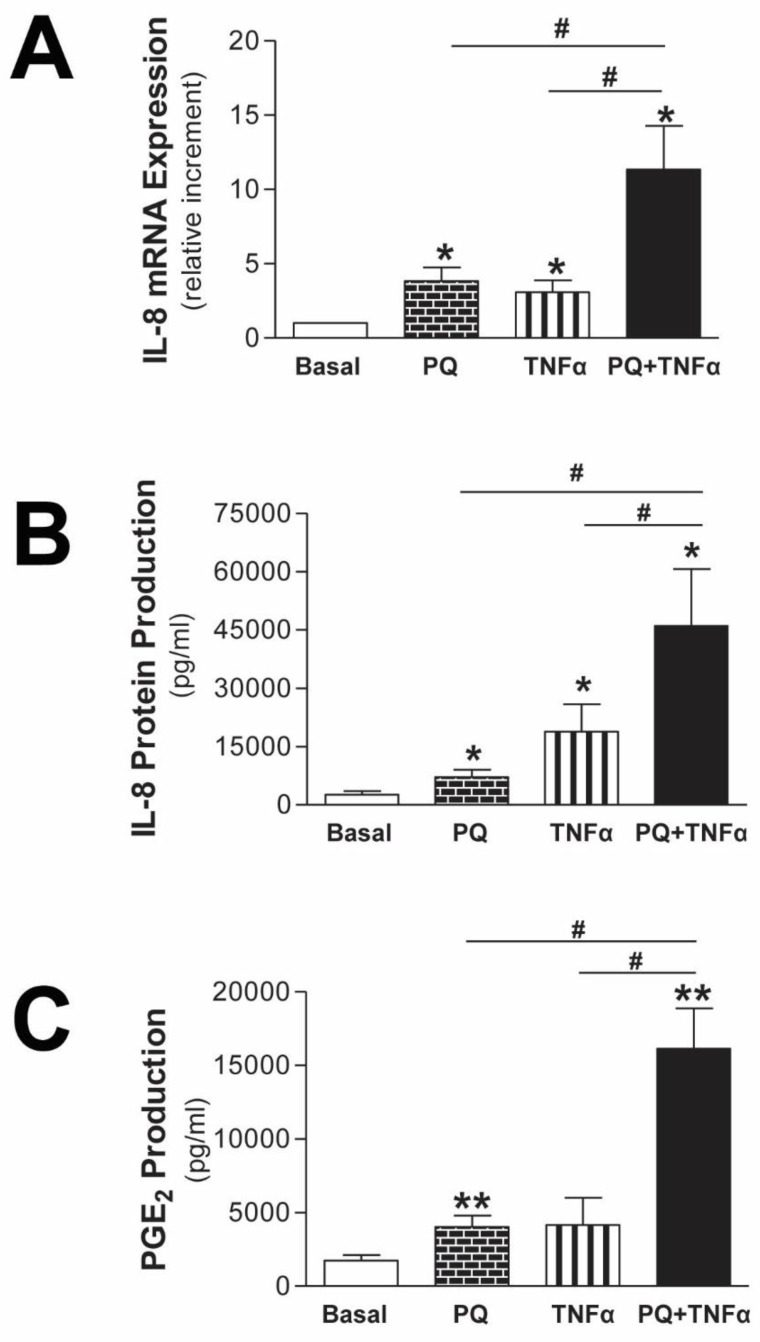
Mitochondrial dysfunction modulates IL-8 and PGE2 response induced by TNFα in human mesothelial cells from peritoneal effluent of PD patients. Mesothelial cells with epithelial phenotype were preincubated with the selective mitochondrial respiratory chain inhibitor paraquat (PQ; 1 mM) for 30 min before treatment with TNFα (1 ng/mL) for 18 h. (**A**) IL-8 mRNA expression is expressed as the mean ± SEM fold change vs. basal condition (n = 7). (**B**) IL-8 protein production is expressed as pg/mL (n = 7). (**C**) The concentration of PGE2 is expressed as pg/mL (n = 5). * *p* ≤ 0.05 and ** *p* ≤ 0.05 vs. basal, and ^#^ *p* ≤ 0.05.

In order to verify this response in primary cells, we followed the same treatment scheme to stimulate mesothelial cells from peritoneal effluent of PD patients with an epithelial phenotype. As a result, ex vivo experiments showed that mitochondrial damage caused by paraquat provokes a synergistic increase in IL-8 mRNA expression induced by TNFα (Figure 3A). As expected, the same result was obtained when IL-8 protein release was determined by ELISA (Figure 3B). The metabolic end product of COX-2, PGE_2_, is also a major player in peritoneal membrane alterations. We also showed in mesothelial cells that sensitizing these cells with paraquat caused a significant increase in PGE_2_ release induced by TNFα (Figure 3C).

All together, these results show that mitochondrial dysfunction has the capacity to increase the sensitivity of human mesothelial cells to proinflammatory cytokines, aggravating the inflammatory response caused by cytokines. 

### 3.3. Mediators Involved in the Aggravation of the Inflammatory Response by the Impairment of Mitochondrial Function in Human Mesothelial Cells 

**Figure 4:**
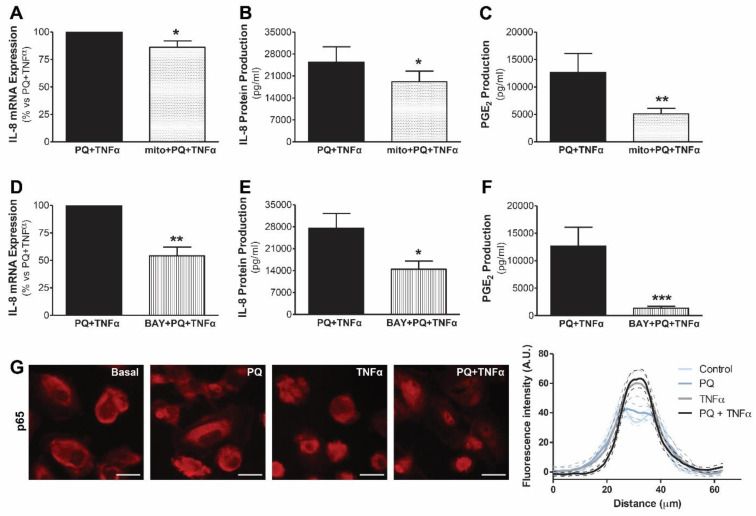
Role of mtROS and NF-κB in the inflammatory response induced by mitochondrial dysfunction in human mesothelial cells from peritoneal effluent of PD patients. Mesothelial cells with epithelial phenotype were preincubated with mitoTEMPO (mito, 10 µM) or BAY 11-7082 (BAY, 10 µM) for 30 min before the treatment with the selective mitochondrial respiratory chain inhibitor paraquat (PQ; 1 mM) and for 30 min plus TNFα (1 ng/mL) for 18 h. (**A**,**D**) IL-8 mRNA expression is expressed as the mean ± SEM fold change vs. control without mitoTEMPO or BAY (n = 13 and n = 5, respectively). (**B**,**E**) IL-8 protein production is expressed as the mean ± SEM pg/mL (n = 21 and n = 14, respectively). (**C**,**F**) PGE_2_ production is expressed as the mean ± SEM pg/mL (n = 17 and n = 11, respectively). (**G**) Representative immunofluorescence of p65 under basal conditions, PQ (40 min), TNFα (20 min) and PQ + TNFα (40 min) (n = 3). Scale bars, 25 µm. Right panel shows the fluorescence intensity plot profile, measured in transects taken through individual cells (n = 30 for each condition). The data were expressed as mean ± SEM. Representative immunofluorescence of p65 under basal conditions, PQ (40 min), TNFα (20 min) and PQ + TNFα (40 min) (n = 3). * *p* ≤ 0.05, ** *p* ≤ 0.01 and *** *p* ≤ 0.001.

Because cumulative oxidative stress caused by mtROS is one of the main causes of high-dialysate glucose-induced human mesothelial cell mitochondrial impairment and having shown in vitro that mitochondrial dysfunction sensitizes mesothelial cells by triggering an inflammatory response to cytokines in human mesothelial cells, we investigated the role of mtROS in this process. As is shown in Figure 4, by inhibiting mtROS production by pre-incubation with the mitochondria-targeted antioxidant mitoTEMPO (10 µM), IL-8 mRNA expression and IL-8 protein release induced by paraquat and TNFα treatment were significantly reduced (*p* ≤ 0.05) in mesothelial cells from the peritoneal effluent of dialysis patients with an epithelial phenotype. Following the same line, mesothelial cells pretreated with mitoTEMPO significantly decreased PGE2 production stimulated by the combination of paraquat and TNFα (*p* ≤ 0.01). Overall, these results suggest the specific involvement of mtROS in the aggravation of the inflammatory response resulting from mitochondrial impairment.

As the transcription factor NF-κB is sensitive to the cellular redox state and regulates numerous genes involved in the modulation of the inflammatory response, we also evaluated its implication in the amplification of the inflammation induced by mitochondrial impairment. For this, mesothelial cells stimulated by the combination of paraquat and TNFα were pretreated with BAY 11-7082 (BAY, 10 µM), a selective inhibitor of NF-κB activation. Our results showed that NF-κB suppression significantly reduced IL-8 mRNA expression and IL-8 and PGE2 production induced by TNFα, in the presence of mitochondrial damage in human mesothelial cells (Figure 4D–F, respectively). When we analyzed NF-κB translocation to the nucleus in mesothelial cells by p65 immunofluorescence (Figure 4G), we found that paraquat-treated mesothelial cells had small amounts of p65 and that the response to TNFα was slightly increased when cells were also preincubated with paraquat.

### 3.4. Resveratrol Protects Mesothelial Cells from Mitochondrial Dysfunction and Inflammatory Response

**Figure 5:**
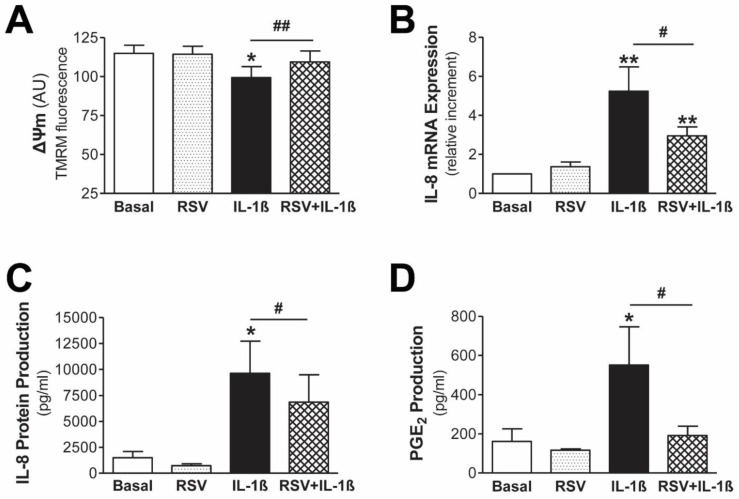
Resveratrol prevents mitochondrial dysfunction and the inflammatory response induced by IL-1β in primary mesothelial cells from PD effluent. Mesothelial cells with an epithelial phenotype were pretreated with resveratrol (RSV; 5 µM) for 30 min before treatment with IL-1β (1 ng/mL). (**A**) Mitochondrial membrane potential was measured by flow cytometry after 12 h of stimulation with IL-1β. Fluorescence quantification for TMRM (n = 7) is expressed as the mean ± SEM of fluorescence intensity in arbitrary units (AU). (**B**) IL-8 mRNA expression after 6 h of stimulation with IL-1β is expressed as the mean ± SEM fold change vs. basal condition (n = 9). (**C**) IL-8 protein (n = 6) and (**D**) PGE2 production (n = 6) were measured by ELISA after 6 h of stimulation with IL-1 β. Data are expressed as mean ± SEM pg/mL. * *p* ≤ 0.05 and ** *p* ≤ 0.01 vs. basal, and ^#^ *p* ≤ 0.05 and ^##^ *p* ≤ 0.01.

Resveratrol is a polyphenol present in our diet, which has been widely recognized for its anti-inflammatory and antioxidant properties. Since the obtained data suggested that mtROS are involved in inflammatory response, we tested the ability of resveratrol to avoid mitochondrial dysfunction induced by IL-1β and to decrease inflammatory response in human mesothelial cells. First, we confirmed that resveratrol is able to prevent the decline in mitochondrial membrane potential caused by IL-1β in human mesothelial cells from PD effluent, as shown by TMRM staining (Figure 5A). Second, pretreatment with resveratrol significantly reduced both mRNA (Figure 5B) and protein (Figure 5C) IL-8 expression, as well as PGE2 production (Figure 5D) induced by IL-1β in mesothelial cells from peritoneal effluent. Resveratrol did not cause any effect on these parameters by itself.

**Figure 6:**
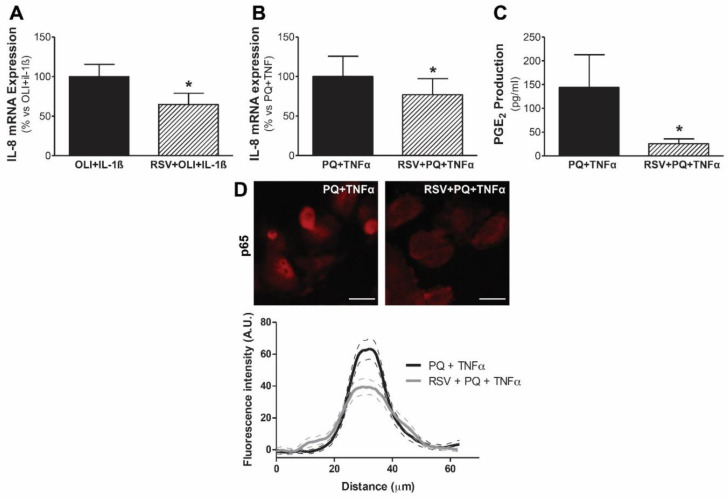
Modulatory effects of resveratrol on the inflammatory response induced by mitochondrial dysfunction plus cytokines. Met5A mesothelial cells were treated with or without resveratrol (RSV; 5 µM) for 30 min before stimulation with oligomycin (OLI; 10 µg/mL) or paraquat (PQ; 1 mM) for 30 min plus treatment with IL-1β (1 ng/mL, 6 h) or TNFα (1 ng/mL, 18 h). IL-8 mRNA expression for (**A**) OLI + IL-1β (n = 7) and (**B**) PQ + TNFα (n = 7). Data are expressed as the mean ± SEM fold change vs. control. (**C**) PGE2 production for PQ + TNFα (n = 10). Data are expressed as mean ± SEM pg/mL. (**D**) Representative immunofluorescence of p65 in Met5A mesothelial cells pretreated or not for 30 min with RSV before PQ + TNFα treatment for 40 min (n = 3). Scale bars, 25 µm. Bottom panel shows the fluorescence intensity plot profile, measured in transects taken through individual cells (n = 30 for each condition). The data were expressed as mean ± SEM.* *p* ≤ 0.05 vs. control.

In addition, treatment with resveratrol also reduced the expression of IL-8 and PGE2 exacerbated by the cytokines IL-1β and TNFα in our model of mitochondrial dysfunction in human mesothelial Met5A cells (Figure 6). In addition, treatment with resveratrol caused a decrease in the nuclear translocation of p65 induced by paraquat plus TNFα. All in all, these results show that resveratrol exerts a protective effect and decreases both the inflammatory response induced by IL-1β and the response enhanced by the synergy between mitochondrial dysfunction and proinflammatory cytokines in human mesothelial cells.

## 4. Discussion

In the last few years, different studies have reported the role of mitochondria in the pathophysiology of peritoneum during PD therapy [13,16,17,19]. In this sense, we have recently demonstrated that mitochondrial dysfunction promotes epithelial-to-mesenchymal transition (EMT) in mesothelial cells of PD patients, activating a process that results in peritoneal fibrosis and progressive loss of the functional characteristics of the membrane [13]. Specifically, we have described a significant increase in mitochondrial reactive oxygen species (mtROS) production and a loss of mitochondrial membrane potential in mesothelial cells with a fibroblast phenotype, compared to those with intact epithelial morphology [13]. Additionally, in agreement with the results obtained in other cohorts, we observed that the level of free mitochondrial DNA (mtDNA) in PD effluent acts as a marker of peritoneal inflammation and also correlates with the functional properties of the peritoneum, so this parameter can help to monitor peritoneal injury in these patients [13,16]. There is also ample evidence for the correlation between the degree of mitochondrial dysfunction and the inflammatory and immune responses [5,11,24], as well as between the inflammatory response, whether acute or chronic, and the progression of fibrosis, including peritoneal fibrosis [25,26,27]. On the other hand, mesothelial cells that coat the peritoneal cavity are directly in contact with dialysis solutions, and play an active role in the disorders of the peritoneum during PD therapy [3,27,28]. The composition of these solutions provokes an inflammatory response in the peritoneum that is measurable by the quantification of cytokines in the PDE. During the first few days of PD, IL-1β concentration is below the detection range in the peritoneal effluent, increasing up to 0.52 ± 0.041 pg/mL on average after 4 months of treatment [29]. These levels become higher when patients suffer from peritonitis. In this case, the median concentration of IL-1β is 52 pg/mL [30], but it can reach values up to 940 pg/mL during infection. Remarkably, it has been described that some bacterial strains, such as Gram-positive strains, are related to higher concentrations of cytokines, such as IL-1β, TNFα and IL-6, in the PDE [31]. We believe that it is of great relevance to extensively study the consequences of this mitochondrial impairment in terms of inflammation and angiogenesis, which could lead to progressive fibrosis of the peritoneal membrane and its consequent functional deterioration. Our present findings on mesothelial cells provide new insights into the complexity of the physiopathology of the peritoneal membrane damage induced by PD that has not been described previously, to our knowledge. In particular, our results indicate that the proinflammatory cytokine IL-1β causes mitochondrial dysfunction in human mesothelial cells from the peritoneal effluent with epithelial phenotypes. In addition, this mitochondrial dysfunction induces a low-grade inflammatory response and sensitizes these cells, boosting their responsiveness to cytokine-induced inflammatory response through the generation of mitochondrial ROS and the activation of the NF-κB pathway. Furthermore, we found that the natural polyphenol resveratrol has the capacity to significantly prevent the inflammatory response.

There is no doubt about the importance of a normal ΔΨm to maintain mitochondrial integrity [5]. Our ex vivo model shows that mitochondria from effluent-derived mesothelial cells with an epithelial phenotype treated with IL-1β suffer from depolarization. Interestingly, an increase in the levels of this cytokine has been reported in the peritoneal effluent of patients during the first month of PD therapy [21]. Together, these facts could explain the significant rise in free mtDNA levels that we found in the effluent of the patients during the first three months of PD treatment, indicating acute mitochondrial damage that could be related with the tissular injury induced by the catheter insertion surgery. In addition, as we described in previous studies, ΔΨm is also altered during the fibrotic process, since mesothelial cells with fibroblastic phenotypes that suffer from EMT show a significant decrease in this parameter, compared to cells with an epithelial phenotype [13]. In this way, almost all the proinflammatory mediators may promote EMT induction [27], which is also stimulated by hyperglycolysis in mesothelial cells, as described by Si et al., who found that this process suppresses mesothelial EMT, preventing the progression of peritoneal fibrosis [32]. Therefore, IL-1β may induce hyperglycolysis in multiple cells [33]. It must be noted that IL-1β can induce elevated superoxide formation via activation of NADPH oxidases, since ROS can be produced not only via the mitochondrial electron transport chain but also via NADPH oxidases [34]. Moreover, a drop in the ΔΨm is a distinguishing mark of apoptotic cell death [35]. In connection with this, other studies have revealed that glucose dialysate induces oxidative stress and mitochondrial-mediated apoptosis, as well as mitochondrial DNA damage in human peritoneal mesothelial cells [17,18]. In addition, it has been shown that the use of more biocompatible dialysis fluids reduces inflammation-induced apoptosis in mesothelial cells exposed to peritoneal dialysis fluid [36]. Altogether, these results consolidate the finding that mitochondrial dysfunction is involved in the development of peritoneal fibrosis. However, future studies are needed to define the precise contribution of cytokines in mitochondrial alteration in mesothelial cells.

In the next set of experiments, we evaluated the potential role of mitochondrial dysfunction in the generation of an inflammatory response in human mesothelial cells. Mitochondrial damage was induced by common mitochondrial inhibitors, such as the mitochondrial pro-oxidant paraquat or the inhibitor of mitochondrial ATP synthase oligomycin [14,15,37]. For this purpose, we focused on IL-8 and COX-2, two key players involved in the inflammatory process that mediates dialysate-induced alterations of the peritoneal membrane [25,38]. In this regard, high levels of IL-8 are present in both the mesothelial cells exposed to PD fluid and effluent of PD patients [39,40]. Our results showed that paraquat significantly increased mesothelial cell IL-8 mRNA expression in a time-dependent manner. When cells were stimulated with oligomycin, an increase was also observed, but of lower intensity, which did not reach statistical significance. Interestingly, this increase was significantly more pronounced when IL-1β was added to mesothelial cells, after the induction of mitochondrial dysfunction. Indeed, in paraquat-treated mesothelial cells, a lower concentration of IL-1β induced a greater response than a five-fold higher concentration of IL-1β in the absence of paraquat pretreatment. It is worth noting that these results are not specific to IL-1β, as TNFα, which is also found at higher levels in PD effluent [16], acts synergistically with mitochondrial dysfunction induced by paraquat, amplifying IL-8 mRNA expression in mesothelial cells. This increase in IL-8 mRNA expression was accompanied by enhanced IL-8 release in effluent-derived mesothelial cells with an epithelial phenotype. New blood vessel formation (angiogenesis) in the peritoneal membrane, which can be regulated by IL-8 [41], plays an indispensable role during the development of peritoneal fibrosis and, in turn, with associated peritoneal ultrafiltration failure progression that is often found in long-term PD patients [26]. In addition, inhibition of peritoneal angiogenesis can significantly decrease peritoneal fibrosis, highlighting its importance in this process and revealing angiogenesis as a potential therapeutic target [42]. On the other hand, IL-8 also reduces mesothelial cell decorin secretion, which has an anti-fibrotic role in peritoneal dialysis [43] and acts as a chemotactic factor that attracts infiltrating inflammatory cells, which have been considered as the main actors responsible for structural and functional disorders of the peritoneum [44].

Another relevant participant in the alterations to the peritoneal membrane is COX-2. In precise terms, its ex vivo expression correlates with the rate of peritoneal transport and is upregulated with the above-mentioned dialysate-induced alterations [25,45]. In addition, it is well-known that the glucose present in PD fluids stimulates the expression of COX-2 [46,47]. Our results showed that mitochondrial dysfunction produced a slight increase in COX-2 expression and production of its metabolic end product, PGE2, in mesothelial cells from patients undergoing PD. Furthermore, we also found that mitochondrial dysfunction aggravated COX-2 expression and PGE2 production, induced by the proinflammatory cytokine TNFα. These findings are consistent with the studies that show that high glucose levels induce mitochondrial damage and PGE2 production in human peritoneal mesothelial cells [17,18,47]. Interestingly, recent data have shown that microsomal prostaglandin E synthase-1 (mPGES-1)-derived PGE2 plays a critical role in PD-associated peritoneal fibrosis through activation of the NLRP3 inflammasome [48], a cytosolic receptor that, once activated, induces the maturation of the proinflammatory cytokines IL-1 and IL-18 [49]. In this sense, there is a positive correlation between the secretion of PGE2 and IL-1β or IL-18 in PD fluid [48]. Furthermore, increased COX-2 expression is a key step to induce pathological angiogenesis [50] and in this manner, it could amplify the local inflammatory process, contributing to the generation of positive feed-back mechanisms that accelerate the inflammatory and angiogenic response. All together, these findings underline the relevance of our results regarding the effects of mitochondrial damage as an aggravator of peritoneal inflammation and angiogenic processes; therefore, mitochondrial integrity preservation could be a novel therapeutic approach to protect the peritoneum from the alterations caused by PD fluids. It is noteworthy that cytokines were used at a higher concentration than that usually found in the PDE to generate the inflammatory response in this study [30]. In this sense, it must be taken into consideration that one of the limitations of this study is the stimulation time, since the MCs that line the peritoneal membrane are exposed to the PDE for months or years. Nevertheless, the time of treatment in in vitro studies is limited.

In a previous ex vivo study, we demonstrated that mesothelial cells from PD effluent with a non-epithelial phenotype produce higher levels of mtROS compared to those with an epithelial phenotype. We also found that mtROS act as mediators of the EMT process that precedes fibrosis in human mesothelial cells [13]. It is important to emphasize the major role that mitochondria play in oxidative stress, being one of the most important sites of ROS production [11]. These mtROS are part of the damage-associated patterns (DAMPS), released by damaged or dysfunctional mitochondria, that are identified by the immune system, and, as a result, can also cause mitochondrial alteration, aggravating and perpetuating a vicious cycle of mitochondrial damage and activation of pathological pathways [5]. Therefore, we focused on evaluating the role of mtROS in the development of the aggravation of the inflammatory response mediated by mitochondrial dysfunction. As a result, when mesothelial cells from patients undergoing PD with epithelial phenotypes were treated with the mitochondria-targeted antioxidant mitoTEMPO, IL-8 mRNA and protein expression, as well as PGE2, induced by the combined treatment of paraquat and TNFα, significantly decreased. These findings agree with in vitro studies on other cell types [15], and also on mesothelial cells, which suggest that TGF-β1also increases the formation of mtROS, leading to an inflammatory scenario, phenotype conversion and, finally, fibrosis [13,51,52,53]. Interestingly, the cumulative oxidative stress caused by mtROS has been revealed as a key factor in mitochondrial impairment induced by glucose-rich PD fluid [17].

One of the key pathways that regulates the inflammatory response is the redox-sensitive transcription factor NF-κB, which controls the mRNA expression of an array of inflammatory mediators, such as the induction of COX-2 and IL-8 expression in multiple cell types, including mesothelial cells [54,55]. In this study, the NF-κB pathway inhibitor BAY significantly prevented the up-regulation of IL-8 mRNA and protein expression and PGE2 production induced by mitochondrial dysfunction, in combination with TNFα. Moreover, damaged mitochondria induced a slight increase in NF-κB activation, evidenced by the increased translocation to the nucleus of p65, which was boosted in response to the exposure to the inflammatory cytokine TNFα, as well as to the combination of PQ and TNFα. Other studies have obtained similar results, reporting enhanced activation of NF-κB under conditions of mitochondrial impairment [15,56,57], but it would be interesting to consider other inflammatory cascades that are further related to mitochondrial damage, such as RIPK, MAPK, ERK, JNK, or JAK-STAT signaling, which could affect this pathway. It is worth noting that IL-8 stimulates the expression of key mediators of angiogenesis, such as VEGF and the autocrine activation of VEGFR2 in endothelial cells by activating NF-κB [58]. In addition, PGE2, the main metabolic product of COX-2, may also provoke a positive vicious cycle, increasing NF-kB downstream signaling [45]. In this manner, NF-κB may contribute to the generation of positive feed-back mechanisms [59].

Currently, there is an active search for natural bioactive compounds that exhibit antioxidant and anti-inflammatory properties for the development of new therapeutic alternatives to modulate the risk of damage suffered by human peritoneal mesothelial cells during PD therapy [60]. Thus, several natural plant compounds have been reported as modulators of EMT transition [60,61]. Resveratrol is a non-flavonoid stilbene polyphenol found in high concentrations in several plants, including grapes, wine, mulberries, peanuts, as well as in Polygonum cuspidatum, whose roots are commonly used in traditional Chinese medicine [61]. Numerous preclinical and clinical analyses have widely recognized its anti-inflammatory, antioxidant, anti-ageing and anti-EMT properties [61,62,63,64]. In the present study, resveratrol was capable of reversing the decline in mitochondrial membrane potential and prevented the increase in the gene expression of IL-8, as well as IL-8 and PGE2 production caused by the proinflammatory cytokine IL-1β in effluent-derived mesothelial cells with an epithelial phenotype. Furthermore, we also found that resveratrol decreased IL-8 and PGE2 expression induced by proinflammatory cytokines and aggravated by mitochondrial dysfunction. This anti-inflammatory effect is mediated by decreased NF-κB activation. In relation to this, the ways in which resveratrol attenuates advanced glycation end product (AGE)-induced damage in human peritoneal mesothelial cells [65] have recently been described, which could potentially lead to mitochondrial damage [66]. Taken together, these data underline the relevance of our findings regarding the beneficial effects of resveratrol to preserve mitochondrial function in mesothelial cells in patients undergoing PD treatment. However, resveratrol, as is the case with other polyphenols, has a relatively low bioavailability. In fact, a maximum peak of 2 µM in human plasma after an intake of 25 mg has been reported [67]. For this reason, it could be of interest to test, in PD patients, the effect of oral intake of this antioxidant, as it has been tested before in a clinical trial with a different final purpose, obtaining promising results [63]. In addition, it would be interesting to investigate its impacts when dissolved in dialysis solutions, in a similar manner to other compounds [68]. In this way, higher concentrations could be reached, achieving a similar dose to the one described in our study, which would be in direct contact with the cells of interest in this case.

Overall, the results of this study provide additional support to the hypothesis that mitochondrial dysfunction plays a role in the inflammatory phenotype of mesothelial cells that leads to their progressive transformation, causing fibrosis development and the consequent functional deterioration of the peritoneal membrane. Mitochondrial damage could induce a low-grade inflammatory response in mesothelial cells and sensitize them to peritoneal environment injury, promoting and perpetuating a vicious cycle of inflammation, which contributes to progressive fibrosis and peritoneal ultrafiltration failure. In this respect, mitochondrial damage can act through modulating innate immunity via redox-sensitive inflammatory pathways or direct activation of inflammasomes [49]. Interestingly, NF-κB via autophagy restricts inflammasome activation through the clearance of damaged mitochondria [69,70]. In addition, resveratrol has been highlighted due to its potential to induce mitophagy, allowing the maintenance of a healthy mitochondrial population and protecting peritoneal mesothelial cells from ROS-NLRP3-mediated inflammatory injury [71]. Future studies should investigate the preservation of mitochondrial function with natural plant compounds as a novel therapeutic approach to prevent peritoneal damage in patients undergoing PD.

## Data Availability

The data presented in this study are available upon request from the corresponding author.

## Supplementary Materials

The following supporting information can be downloaded at: https://www.mdpi.com/article/10.3390/antiox11112184/s1, Figure S1: Mitochondrial dysfunction triggers a low level of inflammatory response in human mesothelial cells.
