# Supplementary material for: Involvement of Mitochondrial Dysfunction in the Inflammatory Response in Human Mesothelial Cells from Peritoneal Dialysis Effluent
Source: Antioxidants (Basel). 2022 Nov 4;11(11):2184. doi: 10.3390/antiox11112184 (PMC9686714; doi:10.3390/antiox11112184)
Supplement: Supplementary file 1 [file antioxidants-11-02184-s001.zip › antioxidants-1973568-supplementary.pdf]

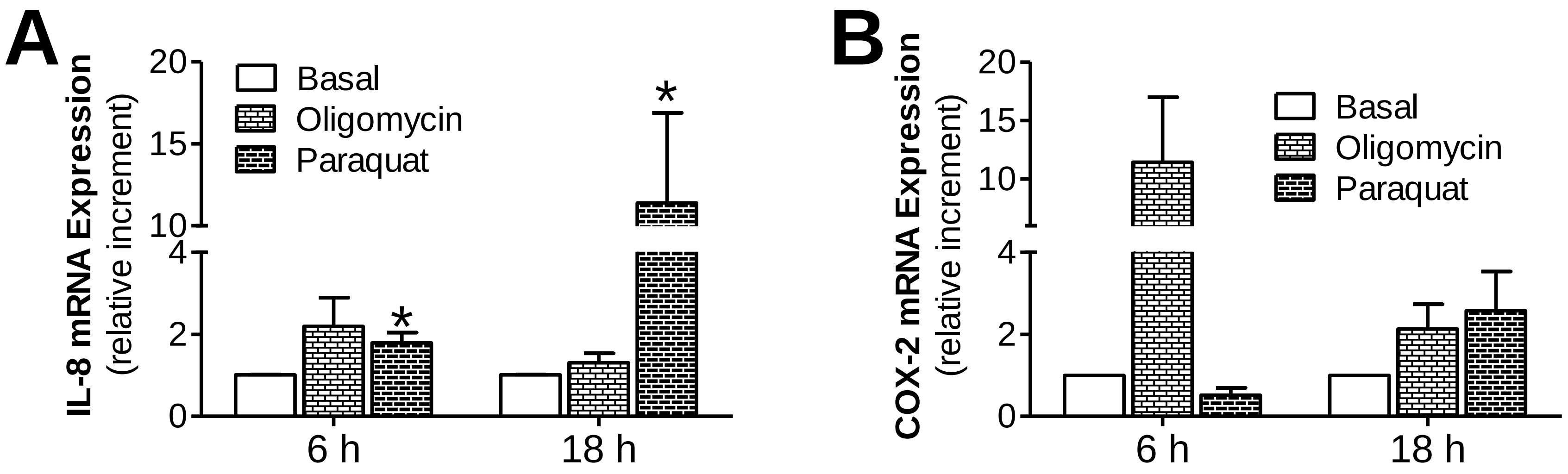

Supplementary Figure S1. Mitochondrial dysfunction triggers a low level of inflammatory response in human mesothelial cells. (**A**) IL-8 and (**B**) COX-2 mRNA expression in Met5A cells incubated under basal conditions or with the selective mitochondrial respiratory chain inhibitors oligomycin (10 µg/ml) or paraquat (1 mM) for 6 and 18 h (n=6 and n=5, respectively). Data are expressed as the mean ± SEM of mRNA expression fold change relative to basal condition. *$P \leq 0.05$ *vs*. basal.
